# Virtual Reality-Based Interventions to Improve Balance in Patients with Traumatic Brain Injury: A Scoping Review

**DOI:** 10.3390/brainsci14050429

**Published:** 2024-04-26

**Authors:** Gabriel Hernan, Neha Ingale, Sujith Somayaji, Akhila Veerubhotla

**Affiliations:** Department of Rehabilitation Medicine, Grossman School of Medicine, New York University, New York, NY 10016, USA; gabriel.hernan@nyu.edu (G.H.); neha.ingale@nyulangone.org (N.I.); sujith.somayaji@nyu.edu (S.S.)

**Keywords:** neurological impairment, balance, falls, virtual reality, brain injury

## Abstract

Introduction: Virtual reality (VR)-based interventions to improve balance and mobility are gaining increasing traction across patient populations. VR-based interventions are believed to be more enjoyable and engaging for patients with traumatic brain injury. This scoping review aims to summarize existing studies from the literature that used VR to improve balance and mobility and determine the gap in VR-based balance literature specific to individuals with traumatic brain injury. Methods: Two authors independently searched the literature using the search terms “Virtual Reality Traumatic Brain Injury Lower Limb”, “Virtual Reality Traumatic Brain Injury Balance”, and “Virtual Reality Traumatic Brain Injury Gait”. Results: A total of seventeen studies, specifically, three randomized controlled trials, one one-arm experimental study, two retrospective studies, two case studies, one feasibility/usability study, one cohort study, and seven diagnostic (validation) studies, met the inclusion criteria for this review. The methodological quality of the studies evaluated using the PEDro scale was fair. Discussion: Future studies should focus on large-scale clinical trials using validated technology to determine its effectiveness and dose–response characteristics. Additionally, standard assessment tools need to be selected and utilized across interventional studies aimed at improving balance and mobility to help compare results between studies.

## 1. Introduction

Each year in the United States, approximately 1.7 million individuals encounter traumatic brain injuries (TBIs) [1]. The immediate impacts of a traumatic brain injury may include unconsciousness of varying length, depression, confusion, trouble recalling the traumatic event or learning new information, speech issues, and lack of coordination [2]. Some or all of the immediate impacts may be permanent [2]. Depending on intrinsic variables like length of unconsciousness and post-traumatic amnesia, TBI is categorized as mild, moderate, or severe [3]. Most people with mild TBI (70–90% of TBI cases) experience rapid recovery, allowing them to reach their pre-TBI health status [4]. Those who experience a TBI with symptoms surpassing three months are considered to have transitioned from the acute to the chronic phase of TBI [5]. Five years post-injury, 57% of moderate or severe chronic TBI patients are moderately or severely disabled, with about 33% relying on others to complete everyday activities [6].

Furthermore, regardless of the extent of severity, health problems due to TBI can cause long-term physical and neurological impairments, affecting the person’s ability to perform daily activities and return to work [7]. About 30–65% of TBI patients report balance impairments sometime during their recovery [8]. Damage to the integration of sensory, motor, and musculoskeletal systems leads to balance issues [9,10]. Impaired balance leads to a higher risk of falls. Falls are the leading cause of TBI-related hospitalizations. Multiple interventions are being developed and evaluated to help improve balance deficits post-TBI. With technological development, research utilizing novel technology to help improve balance deficits post-TBI has been gaining traction over the past decade. 

Traditionally, the standard of care for treating patients with chronic TBI-related balance issues has consisted of various exercises prescribed by a physical therapist, such as firm static standing, foam static standing, and weight-shifting exercises. Physical therapists also use research-based sensory–motor learning concepts available in the clinical setting [11,12]. Physical therapy focusing on sensory stimulation has become a growing trend as rehabilitation techniques modernize [13,14]. In the past decade, the integration of novel Virtual Reality (VR) technology to treat balance issues associated with TBI has grown. VR is a user–computer interface method that incorporates real-time simulation of an environment or activity and permits user input through various sensory channels [15]. Unlike traditional user interfaces, VR allows users to interact with a three-dimensional simulated environment. In VR-integrated rehab, spatial and temporal manipulations are employed to improve sensorimotor training [15]. The physiological activation of brain areas is achieved using VR rehabilitation programs, as it involves motor learning and repeated practice with stimuli from multiple senses (audio, visual, motor, and proprioceptive) [16].

VR-integrated rehab has some benefits over the standard of care. VR rehabilitation allows for precise, objective progress tracking and seems to motivate patients more than traditional rehabilitation practices [17]. Additionally, using VR to treat TBI balance issues allows therapists to control the stimuli and simulate environments tightly without risking patient safety [18]. Finally, VR therapy has also shown greater ecological validity while having the ability to create more affordable environments that can be reused by other therapists [19].

Research involving VR is broadly classified into three main categories—immersive, non-immersive, and semi-immersive VR. The immersive VR creates a 360-degree environment with a headset or goggles that makes the user feel as though they are inside the virtual environment. Second is the non-immersive VR, which displays content on a device such as a television, computer screen, or any other surface and allows the user to see the computer-generated environment on the screen [20]. Semi-immersive VR is a mixture of immersive and non-immersive VR, allowing users to interact with the virtual environment while physically connecting to their surroundings. The advancement in technology and reduction in cost has allowed these VR devices to expand in scope and thus become widespread in research [21]. While extensive research exists on utilizing VR for upper extremity rehabilitation, research implementing VR to help improve gait and balance deficiencies post-TBI is relatively new. Although systematic reviews summarizing VR interventions on traumatic brain injuries exist, most have focused only on upper extremity rehabilitation. Only one study focused on reviewing five randomized controlled trials (RCT) related to lower extremity VR rehabilitation [22]. A full scoping review of the literature to understand the existing role of VR in balance research post-TBI is needed to determine research gaps and future research directions [22]. This review aims to fill the gap in the literature by completing a full scoping review of VR interventions aimed at improving balance and mobility deficits in adults with TBI. This review will summarize existing literature related to lower limb VR rehabilitation. This review is essential as a starting point in guiding clinical practice. This review will highlight existing gaps in the literature and evoke new research ideas.

## 2. Materials and Methods

A scoping review was conducted. Research papers published between 2016 and 2023 were identified from *Google Scholar*, *PubMed*, *Science Direct*, *Web of Science*, *Scopus*, and *Cochrane Library* databases. The search phrases used were as follows: “Virtual Reality Traumatic Brain Injury Lower Limb”, “Virtual Reality Traumatic Brain Injury Balance”, and “Virtual Reality Traumatic Brain Injury Gait”. Two authors (GH and NI) independently conducted the search and filtered papers based on their titles and abstracts, finding 36 initial papers.

The following inclusion criteria were used: (a) VR was used as a therapy, (b) this therapy focused on balance dysfunction, and (c) at least one patient with a traumatic brain injury was included in the study. The following exclusion criteria were used: (a) the study primarily included children, (b) the study included only healthy older adults, or (c) it was a systematic review.

**Figure 1:**
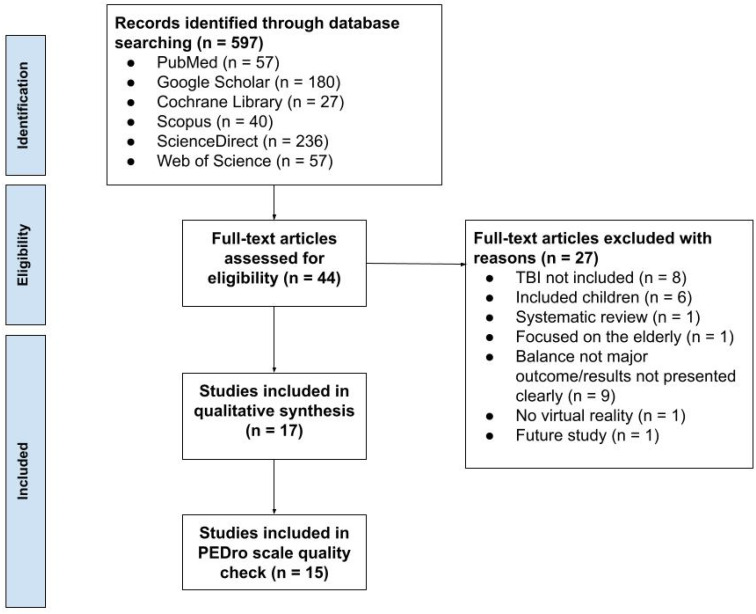
PRISMA study flow chart.

Initially, 36 papers were independently found by two authors (GH and NI) based on their titles and abstracts. The two authors independently read the full text of the initial 36 papers and met to discuss the search results based on the inclusion and exclusion criteria. After meeting and discussing the papers found, 17 were selected that met the inclusion and exclusion criteria. The nineteen papers that were removed did not match the study criteria: five did not include any TBI patients, six included only children, one was a systematic review, one focused on healthy older adults, and six did not report balance as their major outcome. The study followed PRISMA guidelines, and the PRISMA flow diagram is presented in Figure 1.

Then, the two authors independently evaluated the methodological quality of the clinical trials using the PEDro scale [23], while the case–control studies were evaluated using the Joanna Briggs Institute Critical Appraisal tool. The PEDro scale assesses external validity, internal validity, and statistical reporting through an 11-item checklist. A score is calculated out of 10 by giving the items a score of 1 or 0; scores <4 are deemed “poor”, 4–5 are deemed “fair”, 6–8 are deemed “good”, and 9–10 are deemed “excellent” [23]. Once both authors scored the papers independently, they met again to compare the scores. Individual scores were compared, and any discrepancies were resolved through discussion. Two papers were excluded from the PEDro scale quality check because they were case studies that the PEDro scale is not designed to evaluate (Figure 1). The Joanna Briggs Institute Critical Appraisal tool for case studies is a ten-questionnaire scale that assesses internal validity and risk of bias of case series designs, particularly confounding, selection, and information bias, in addition to the importance of clear and transparent reporting [23]. The 17 papers included in this review were organized into an Excel sheet for further analysis. They were divided into categories such as randomized controlled trials (RCTs), case studies, feasibility or usability studies, validation studies, etc., based on the type of research paper. 

## 3. Results

Numerous study design types were identified: three randomized controlled trials, one one-arm experimental study, two retrospective studies, two case studies, one feasibility/usability study, one cohort study, and seven diagnostic (validation) studies..

### 3.1. Risk of Bias Analysis

The average PEDro scale score was 4.8, with a standard deviation of 1.6. Based on the grading scale described in the methods section, the papers were graded as follows: “poor” = 4 studies (26.7%), “fair” = 8 studies (53.3%), and “good” = 3 studies (20.0%). No papers received an “excellent” grade. Based on the score breakdown, it can be concluded that the overall methodological quality of the studies was fair. The PEDro scale detailed evaluation of all studies is presented in Table 1. Detailed PEDro scale analysis for studies is provided in Supplementary Materials.

The two case studies scored satisfactorily on the Joanna Briggs Institute Critical Appraisal tool, with their only limitation being that neither of the case studies reported any information on adverse or unanticipated events.

Data extracted from all papers in each category are summarized in Table 2. Demographic data from all studies is summarized in Table 3.

### 3.2. Randomized Controlled Trials

These studies included an average of 38.0 (SD = 22.3, range = 20–63) TBI patients. Two studies utilized the Xbox Kinect device (Microsoft, Redmond, WA, USA), and the third study used the Motek C-Mill™ treadmill (Motek, Amsterdam, The Netherlands). All three VR systems are considered non-immersive. One study aimed to compare the safety and effectiveness of virtual reality-based treadmill training with treadmill training alone and the standard of care. This study took baseline measurements followed by 12 training sessions over a four-week period, with post-treatment measurements taken within one week of the final session and follow-up measurements taken four weeks after the final training session. The VR + Treadmill, Treadmill, and Standard of Care groups saw improvements from baseline to post-treatment of 5.9 (33.1 to 39.0), 5.1 (27.4 to 32.5), and 6.2 (31.6 to 37.8), respectively [24]. All three groups had similar improvements on the Community Balance and Mobility Scale (CB&M). The VR + Treadmill Training group retained improvement via the follow-up, while the Treadmill Training and the Standard of Care groups improved further at the follow-up by 3.4 points. Another study aimed to assess the efficacy of a 12-week home VR-based intervention program and compare it with a Home Exercise Program (HEP) with respect to balance. The intervention consisted of three to four 30 min weekly training sessions for 12 weeks, with follow-up measurements taken after 24 weeks. This study concluded that the VR training did not improve balance to a greater extent than the traditional home exercise program (HEP), but both treatment groups improved from their baseline scores [25]. The VR group improved its CB&M score from baseline to 12 weeks by 7.73 (SE = 1.66, 95% CI = 4.41–11.05), and the HEP increased its score by 7.87 (SE = 1.66, 95% CI = 4.55–11.19). The final study’s aim was to compare balance improvement after Video Game Therapy (VGT) or Balance Platform therapy (BPT). The intervention lasted for six weeks with one-hour sessions three times per week. This study found that the VGT and BPT groups improved on the Unified Balance Scale (UBS) by 6.5 (43 to 49.5) and by 2 (from 49 to 51) points, respectively [26]. Only the Video Game Therapy group significantly improved on the CB&M with a score increase from pre-treatment to post-treatment of 8 (from 17 at baseline to 25 at post-treatment).

### 3.3. Single-Arm Experimental Study

The aim of this study was to quantify the shift in sensorimotor control and measure the injury rate. The study included 28 TBI and 44 non-TBI patients and involved two face-to-face sessions per week (45 min each) for four weeks and a Home Exercise Program (HEP). It used the Oculus Go VR Headset (Meta, Menlo Park, CA, USA), an immersive system [27]. The TBI group improved their Static Balance (Sway Score) from 88.6 to 93.7 (*p* < 0.001), while the non-TBI group improved their sway score from 88.2 to 91.2 (*p* = 0.006).

### 3.4. Retrospective Studies

One study reported on balance and gait and VR-based rehabilitation used in clinical practice. The VR system utilized was the Computer Assisted Rehabilitation Environment (CAREN) High-End (Motek, Amsterdam, Netherlands), the CAREN Base, the V-Gait (Motek, Amsterdam, Netherlands), and the C-Mill which are all non-immersive VR systems [28]. The intervention consisted of one pre-treatment assessment session and 11 training sessions lasting 30–45 min each, with a reassessment after session 12. The study reported on multiple patient categories and separated the results by patient category, including 10 total TBI patients. However, not all TBI patients completed every test. There was an improvement on the Mini Balance Evaluation Systems Test (Mini BESTest) (six patients), the Berg Balance Scale (BBS) (five patients), the Timed up and Go Dual Task (TUG-DT) (five patients), and the Four Square Step Test (FSST) (two patients). Five patients did not improve on the 10-Meter Walk Test (10 MWT). Another study examined the effects of a VR system for balance training on hemiplegic patients with neurological impairments [29]. The intervention consisted of two sessions per week for eight weeks, with a total of 15 training sessions. This study included 6 TBI, 29 stroke, and 6 tumor patients but did not divide the results by patient population [29]. The study used the Nintendo Wii Fit system (Nintendo, Kyoto, Japan) (non-immersive). There was a significant improvement in the BBS for both the intervention and control groups. There was also a significant difference between the two groups (*p* < 0.001) for the BBS and Activities-Specific Balance Confidence Scale (ABC).

### 3.5. Case Studies

One study assessed the effects of Kinetic-based VR intervention on balance outcomes for TBI patients. VR system utilized was the Xbox One^®^ and Kinect^®^ sensor (Microsoft, USA) and a 45” television (Non-Immersive) [30]. The study consisted of a 12-week baseline phase, an eight-week intervention phase, and a four-week retention period. Dynamic Gait Index (DGI) improved during the intervention phase (from 11.8 at baseline to 16.2), and static balance did not significantly change based on the Functional Reach Test (FRT). Another study examined using VR-based real-time feedback for gait training affects motor functions and gait abilities. This study used a Quasar® Med treadmill (H/P/Cosmos, Nussdorf am Inn, Germany), Oculus Rift VR device (Meta, Menlo Park, CA, USA), and smart insoles (R-C-SPO-Pedisol250, SPINA Systems Co., Ltd., Gyeonggi-Do, Republic of Korea), which constitute an immersive VR system [31]. This study consisted of five sessions per week for eight weeks, with training sessions consisting of 20 min of VR-based training and 30 min of general physical therapy. The Center of Pressure (COP) decreased from 35.62 cm at pre-test to 32.67 cm, and the Limits of Stability (LOS) increased post-intervention. The patient’s gait ability improved, and his activity function improved with his Fugl-Meyer Assessment (FMA) score increasing from 18 to 23.

### 3.6. Feasibility/Usability Study 

One study evaluated the VR application that used the Head-Mounted Display (HMD) to target dizziness and sensory integration in patients. This study included two TBI, one vestibular migraine, and 12 unilateral peripheral hypofunction patients. The study utilized the HTC Vive VR system (HTC, New Taipei City, Taiwan) (immersive) with an average of six sessions (SD = 1.3), with a maximum of eight and a minimum of three sessions [32]. The TBI patients improved on the 8-foot up and go (8FUG), with their average time decreasing from 6.85 to 5.94 s. The results for the ABC and Visual Vertigo Analogue Scale (VVAS) could not be analyzed because they were not presented in the correct format.

### 3.7. Cohort Study

The aim of this study was to determine if stress reactivity and postural control are susceptible to long-term consequences of mild traumatic brain injury (mTBI). The study included 14 TBI (13 for virtual environment TBI screening) and 22 with No History of TBI (19 for Virtual Environment TBI Screening) subjects [33]. The study utilized the Wii Balance Board (Nintendo, Kyoto, Japan) and a 60 in. (75 cm high × 134 cm wide) television (non-immersive) for one session. Participants with more than one mTBI produced the greatest COP sway area on Dynamic Foam (DYN-Foam), and participants with no mTBI history produced greater COP sway area on Eyes Closed Foam (EC-Foam), Eyes Open Foam (EO-Foam), and EO, EC, DYN Firm.

### 3.8. Diagnostic (Validation) Studies

One study used the CAREN system with the aim to differentiate between TBI and healthy subjects. The study consisted of one session with 1.5 h of clinical tests and two hours using the CAREN system [34]. The other study using the CAREN system aimed to determine physical performance during VR tasks; three used the Wii Balance Board with a television to determine statistical group differences between healthy and concussed participants, investigate the role of visual-vestibular processing deficits and extend our understanding of the connection between the endorsement of symptoms and the identification of signals associated with balance dysfunction. One study used the Head Rehab VR System to evaluate a VR balance module’s sensitivity and specificity to identify persistent balance deficiencies. One study used the BioVRSea system to verify the classification of concussions versus non-concussions and statistically evaluate the various physiological reactions during postural control activities linked to concussion symptoms. The average number of TBI patients was 47.7 (SD = 73.8, range = 11–214), and the average number of non-TBI patients was 44.3 (SD = 31.1, range = 10–94). One study [34] utilized Sensorimotor Perturbations (standing and walking) to identify whether participants had an mTBI. The standing perturbations had an average accuracy ≈ 0.65 and the walking perturbations had an average accuracy ≈ 0.90. Study [35] found that the virtual environment TBI screen identified TBI participants with a 91.0% accuracy. The screen also had a ROC curve with AUC = 0.865, *p* < 0.001. The Neurocom Sensory Organization Test had 84.0% accuracy (AUC = 0.703, *p* = 0.034). Study [36] evaluated the VR balance module, which had a sensitivity of 85.7% and a specificity of 87.8% (cutoff score = 8.25) for identifying patients with TBI. The AUC was 0.862 (95% CI; 0.767–0.958). Study [37] used a single forward conditional regression model combining DYN-foam, optokinetic, horizontal eye saccades, and the convergence test to reach 94.4% accuracy (AUC = 0.998, *p* < 0.001) in identifying TBI participants. The model had 100% sensitivity and a specificity of 93.1%. The BESS score was not correlated with health status. Study [38] found that the mTBI^+^ group had a greater COP sway area with DYN-Foam than the mTBI^−^ group. Across all Virtual Environment TBI Screening (VETS) conditions, the mTBI^1+^ group generally had increased COP sway compared to the mTBI^1^ and mTBI^−^ groups. One study [39], which consisted of a four-week program, found that The Balance Balls environment had an AUC = 0.618 (*p* = 0.007) for identifying TBI participants. The Balance Cubes—Static virtual environment had an AUC = 0.664 (*p* < 0.001), and the Balance Cubes—PM virtual environment had an AUC = 0.688 (*p* < 0.001). Study [40] created a system by using machine learning and combining SCAT5 with BioVRSea parameters that can classify concussion and non-concussion with an accuracy of up to 95.5%.

**Table 1:** Methodological quality of studies according to the PEDro scale.

Title	Author	Date Published	Score
Feasibility of virtual reality and treadmill training in traumatic brain injury: a randomized controlled pilot trial [24]	Tefertiller et al.	2022	8
Sensorimotor conflict tests in an immersive virtual environment reveal subclinical impairments in mild traumatic brain injury [25]	Rao et al.	2020	5
Results From a Randomized Controlled Trial to Address Balance Deficits After Traumatic Brain Injury [26]	Tefertiller et al.	2019	8
Assessing subacute mild traumatic brain injury with a portable virtual reality balance device [27]	Wright et al.	2016	5
Differential Sensitivity Between a Virtual Reality Balance Module and Clinically Used Concussion Balance Modalities [28]	Teel et al.	2016	4
Visual-vestibular processing deficits in mild traumatic brain injury [29]	Wright et al.	2017	4
Advanced virtual reality-based rehabilitation of balance and gait in clinical practice [32]	Porras et al.	2019	3
History of Mild Traumatic Brain Injury Affects Static Balance under Complex Multisensory Manipulations [33]	Wright et al.	2022	5
Expanding Clinical Assessment for Traumatic Brain Injury and Comorbid Post-Traumatic Stress Disorder: A Retrospective Analysis of Virtual Environment Tasks in the Computer-Assisted Rehabilitation Environment [34]	Onakomaiya et al.	2017	5
Healthy Active Duty Military with Lifetime Experience of Mild Traumatic Brain Injury Exhibits Subtle Deficits in Sensory Reactivity and Sensory Integration During Static Balance [35]	Wright et al.	2018	3
Contextual sensory integration training via head mounted display for individuals with vestibular disorders: a feasibility study [36]	Lubetzky et al.	2022	3
Sensorimotor training for injury prevention in collegiate soccer players: An experimental study [37]	Reneker et al.	2019	3
The effects of video game therapy on balance and attention in chronic ambulatory traumatic brain injury: an exploratory study [38]	Straudi et al.	2017	7
Effects of Balance Training Using a Virtual Reality Program in Hemiplegic Patients [39]	Jung-Ah Kwon, Yoon-Kyum Shin, Deok-Ju Kim, Sung-Rae Cho	2022	5
Towards defining biomarkers to evaluate concussions using virtual reality and a moving platform (BioVRSea) [40]	Jacob et al.	2022	4

**Table 2:** (**a**). Randomized controlled trial. (**b**). One-arm experimental study. (**c**). Retrospective study. (**d**). Case study. (**e**). Feasibility/usability study and cohort study. (**f**) Diagnostic (validation study).

(**a**)													
**Sr.** **No**	**Study Duration**	**VR System**	**Outcomes**		**Participant Groups**								
[24]	1 baseline; 12 training sessions over 4 weeks (after 2 weeks of baseline); 1 post-treatment assessment (within 1 week of final training session), follow up assessment (after 4 weeks of final training session)	Motek C-Mill™ treadmill (Non-Immersive)	**Assessment**	**Metric**	**Virtual Reality + Treadmill Training** **N = 10**			**Treadmill Training** **N = 11**			**Standard of Care** **N = 10**		
			Community Balance and Mobility Scale	Mean (SD)	B	PT	F*	B	PT	F*	B	PT	F*
					33.1 (21.3)	39.0 (24.6)	39.0 (24.2)	27.4 (25.1)	32.5 (27.7)	35.9 (28.5)	31.6 (21.5)	37.8 (25.6)	41.2 (25.6)
			10 Meter Walk Test (Speed, meters/second	Mean (SD)	1.09 (0.45)	1.21 (0.49)	1.14 (0.38)	1.05 (0.50)	1.12 (0.47)	1.11 (0.45)	0.99 (0.44)	1.03 (0.46)	1.02 (0.38)
			6 Minute Walk Test (Distance, meters)	Mean (SD)	344.0 (122.4)	377.4 (151.8)	398.2 (138.5)	359.2 (157.1)	397.3 (180.7)	402.9 (166.4)	343.7 (149.2)	374.5 (151.3)	378.3 (154.4)
			Timed Up and Go Test (Time, seconds)	Mean (SD)	16.4 (14.6)	14.3 (10.4)	13.4 (7.0)	17.7 (13.3)	17.2 (13.3)	16.6 (13.7)	21.5 (30.1)	17.5 (21.3)	19.9 (26.4)
			Physical Activity Enjoyment Scale	Mean (SD)	105.3 (16.2)	113.9 (12.7)	N/A	111.7 (14.5)	112.9 (16.1)	N/A	103.9 (17.3)	103.3 (19.8)	N/A
[25]	3–4 training sessions/week (30 min) for 12 weeks; 1 follow-up on week 24	X-Box Kinect (Non-Immersive)	Outcomes		**TBI (VR Group)**				**TBI (Home Exercise Program Group)**				
			Assessment	Metric	6 weeks*	12 weeks*	24 weeks*		6 weeks*	12 weeks*		24 weeks*	
			Community Balance and Mobility Scale	Mean Estimate	5.19	7.73	8.60		5.49	7.87		8.73	
				SE	1.31	1.66	1.39		1.31	1.66		1.37	
				95% CI	2.57–7.81	4.41–11.05	5.81–11.38		2.87–8.11	4.55–11.19		5.99–11.48	
				*p* Value	0.0002	<0.0001	<0.0001		<0.0001	<0.0001		<0.0001	
			Balance Evaluation System Test (BESTest) Changes from Baseline	Mean Estimate	3.90	5.27	6.80		3.89	5.36		5.89	
				SE	1.31	1.69	1.44		1.31	1.69		1.42	
				95% CI	1.28–6.52	1.89–8.65	3.92–9.68		1.27–6.51	1.99–8.74		3.05–8.74	
				*p* Value	0.0042	0.0028	<0.0001		0.0043	0.0023		0.0001	
			Activities-Specific Balance Confidence Scale (ABC)	Mean Estimate	3.30	1.62	3.75		0.65	2.60		2.45	
				SE	1.76	1.64	1.91		1.75	1.64		1.64	
				95% CI	−0.23 to 6.82	−1.66 to 4.90	−0.08 to 7.57		−2.86 to 4.16	−0.67 to 5.88		−0.67 to 5.88	
				*p* Value	0.0663	0.3271	0.0550		0.7138	0.1171		0.1171	
			Participation Assessment with Recombined Tools-Objective (PART-O)	Mean Estimate	0.00	0.02	0.07		0.08	0.04		0.04	
				SE	=0.05	0.05	0.07		0.05	0.05		0.07	
				95% CI	−0.11 to 0.10	−0.09 to 0.13	−0.08 to 0.21		−0.03 to 0.19	−0.07 to 0.14		−0.11 to 0.18	
				*p* Value	0.9523	0.7023	0.3676		0.1494	0.4977		0.6204	
[26]	3 sessions/week for 6 weeks (1 h)	Xbox 360 Kinect (Non-Immersive)	Outcomes		**TBI (VGT)**				**TBI (BPT)**				
			Assessment	Metric	B**		PT**		B**		PT**		
			Community Balance and Mobility Scale	Median (IQR)	17 (15)		25 (15.5)		25 (32)		25.5 (31.5)		
			Unified Balance Scale	(UBS)	43 (20.5)		49.5 (20.5)		49 (18.5)		51 (20.5)		
			Timed up and Go Test	(Time, seconds)	18.7 (16.1)		16.4 (9.4)		14.0 (20.3)		15.4 (16.2)		
			Static balance	ML path length (mm)	EO: 154.9 (56.0) EC: 161.2 (68.3)		EO: 140.7 (83.9) EC: 188.1 (85.0)		EO: 169.5 (539.5) EC: 218.8 (508.3)		EO: 201.0 (128.3) EC: 233.5 (145.8)		
				AP path length (mm)	EO: 223.7 (80.9) EC: 312.0 (141.1)		171.2 (137.6) EC: 311.3 (147.9)		EO: 258.3 (127.6) EC: 332.5 (419.6)		EO: 262.7 (226.1) EC: 321.6 (480.4)		
				Sway speed (mm/s)	EO: 15.6 (6.9) EC: 19.2(4.3)		12.7 (8.6) EC: 19.7 (10.1)		EO: 18.2 (24.4) EC: 22.9 (35.8)		EO: 20.9 (9.8) EC: 23.5 (22.8)		
				Tot path length (mm)	EO: 309.5 (137.0) EC: 382.0 (85.6)		252.1 (170.7) EC: 392.0 (201.6)		EO: 362.0 (486.4) EC: 456.3 (714.3)		EO: 416.3 (194.8) EC: 468.5 (454.5)		
			Selective visual attention evaluation (Go/No go task reaction	time (ms)	569.5 (205)		557 (179)		568 (146)		576 (166)		
(**b**)													
**Sr.** **No**	**Study Duration**		**VR System**		**Outcomes**		**Participant groups**						
[27]	2 face-to-face sessions per week (45 min. each) for 4 weeks and a home exercise program (HEP)		Headset VR (Oculus Go) (Immersive)		Assessment	Metric	**TBI**				**Non-TBI**		
							B*		PT (after session 8)		B	PT (after session 8)	
					Static balance	Sway score	88.6		93.7		88.2	91.2	
						*p* value	N/A		<0.001		N/A	0.006	
(**c**)													
**Sr.** **No**	**Study Duration**		**VR System**		**Outcomes**		**Participant groups**						
[28]	1 full assessment session (PRE) and 11 tailored training sessions (30–45 min each); Reassessment after session 12 (POST)		Computer Assisted Rehabilitation Environment (CAREN) High-End, the CAREN Base, the V-Gait, the C-Mill (Non-Immersive))		Assessment	Metric	TBI						
							B*				PT (after session 12):		
					Mini BESTest	mean ± error	14.83 ± 2.21				15.5 ± 2.83		
						N	6				6		
					Berg Balance Scale	mean ± error	26.00 ± 5.94				29.00 ± 9.55		
						N	5				5		
					10 Meter Walk Test-DT (10MWT)	(mean ± error)	0.76 ± 0.14				0.75 ± 0.10		
						N	5				5		
					Timed Up and Go-DT (TUG)	mean ± error	18.52 ± 3.21				17.16 ± 1.97		
						N	5				5		
					Four Square Step Test (FSST)	mean ± error	19.13 ± 6.32				15.36 ± 4.87		
						N	2				2		
[29]	Total 15 training sessions (2x/week for 8 weeks)		Nintendo Wii Fit (Non-Immersive)		Outcomes		TBI, stroke, tumor (Intervention group)				TBI, stroke, tumor (control group)		
					Assessment	Metric	B*		PT (after session 15)		B*	PT (after session 15)	
					Berg Balance Scale (BBS)	Score	42.10 +/− 9.36		48.10 +/− 7.18		47.00 +/− 8.52	48.35 +/− 7.71	
					10-Meter Walk Test (10MWT) Walking Speed (m/s)	Speed (m/s)	Regular speed: 1.54 +/− 0.50 Fast speed: 1.21 +/− 0.50		Regular speed: 1.29 +/− 0.41 Fast speed: 1.06 +/− 0.40		Regular speed: 1.30 +/− 0.51 Fast speed: 1.03 +/− 0.38	Regular Speed: 1.25 ± 0.54 Fast Speed: 0.96 ± 0.32	
					Activity-Specific Balance Confidence (ABC)	Score	55.95 +/− 22.74		69.76 +/− 20.98		64.99 ± 29.82	66.10 ± 27.87	
(**d**)													
**Sr.** **No**	**Study Duration**		**VR System**		**Outcomes**		**Participant groups**						
[30]	Baseline phase (12 weeks); Intervention phase (8 weeks); Retention period (4 weeks)		Xbox One^®^ and Kinect^®^ sensor (Microsoft, Redmond WA, USA), 45″ Samsung television (Non-Immersive)		Assessment	Metric	TBI						
							B*		I*		R*		
					Limits of Stability (LOS) (end-point excursion (EPE))	(Mean and SD)	Front: 67.3 (SD = 10.2) Right: 69.4 (SD = 6.1) Back: 74.1 (SD = 5.4) Left: 75.0 (SD = 5.7)		Front: 69.9 (SD = 6.5) Right: 70.5 (SD = 8.6) Back: 84.6 (SD = 13.0) Left: 79.3 (SD = 12.1)		Front: 80.2 (SD = 10.5) Right: 70.0 (SD = 2.9) Back: 78.1 (SD = 9.6) Left: 73.5 (SD = 6.8)		
					Limits of Stability (LOS) (maximal excursion (MXE))	(Mean and SD)	Front: 98.5 (SD = 8.6) Right: 94.2 (SD = 6.9) Back: 100.1 (SD = 7.6) Left: 99.8 (SD = 8.5)		Front: 99.7 (SD = 5.3) Right: 98.7 (SD = 6.3) Back: 108.3 (SD = 7.2) Left: 101.8 (SD = 7.8)		Front: 107.0 (SD = 3.8) Right: 100.4 (SD = 2.9) Back: 106.6 (SD = 10.7) Left: 107.0 (SD = 6.7)		
					Limits of Stability (LOS) directional control (DCL) (Mean and SD)	(Mean and SD)	Front: 67.33 (SD = 5.8) Right: 55.02 (SD = 11.3) Back: 50.5 (SD = 8.6) Left: 68.3 (SD = 4.3)		Front: 74.2 (SD = 4.8) Right: 66.8 (SD = 8.6) Back: 59.1 (SD = 3.0) Left: 73.1 (SD = 3.6)		Front: 67.5 (SD = 7.5) Right: 64.2 (SD = 9.4) Back: 60.5 (SD = 10.4) Left: 68.5 (SD = 4.5)		
					Functional reach test (FRT)	(Mean and SD)	Condition 1 Both palms: 28.9 (SD = 2.9) Condition 2 Left palm: 37.0 (SD = 2.3) Condition 3 Right palm: 36.6 (SD = 1.9)		Condition 1 Both palms: 28.9 (SD = 1.9) Condition 2 Left palm: 36.8 (SD = 1.0) Condition 3 Right palm: 37.5 (SD = 2.0)		Condition 1 Both palms: 26.9 (SD = 1.6) Condition 2 Left palm: 34.7 (SD = 1.4) Condition 3 Right palm: 36.0 (SD = 2.0)		
					Dynamic Gait Index (DGI)	(Mean and SD)	11.8 (SD = 0.4)		16.2 (SD = 2.3)		19 (SD = 0.0)		
[31]	20 min. of VR-based training and 30 min. of general physical therapy (5×week for 8 weeks)		Treadmill (Quasar Med, Nussdorf am Inn, Germany), Oculus Rift VR device, smart insoles (R-C-SPO-Pedisol250, Pedisol, Korea) (Immersive)		Assessment	Metric	TBI						
							Baseline				PT (8 Weeks)		
					BioRescue	COP and LOS	COP: 35.62 cm LOS: 6625.62 cm^2^				COP: 32.67 cm LOS: 7123.52 cm^2^		
					Gait Ability	measured by GAITRite	ASL (cm): 32.96 SL (cm): 67.66 ASS (%): 25.67 Cadence (step/second): 72				ASL (cm): 41.59 SL (cm): 75.12 ASS (%): 32.12 Cadence (step/second): 82		
					Activity Function	measured by Fugl–Meyer Assessment (FMA)	FMA: 18				FMA: 23		
(**e**)													
**Sr.** **No**	**Study Duration**		**VR System**		**Outcomes**		**Participant groups**						
[32]	Average of 6 sessions (SD = 1.3); Maximum = 8; minimum = 3.		HTC Vive (Immersive)		Assessment	Metric	TBI						
							B*				PT*		
					8-foot up and go (8FUG)	(Mean)	6.85 s				5.94 s		
					Activities-Specific Balance Confidence	(ABC) Scale (%)	Improvement on the ABC scale between B* and PT* was 8.3 (SD = 9.03)%						
					Visual Vertigo Analogue Scale	(VVAS) cm	Improvement on the VVAS scale between B* and PT* was 19.8 (SD = 25.03)cm						
Cohort study													
[33]	One session		Wii Balance Board (WBB), 60 in. (75 cm high × 134 cm wide) television (Non-Immersive)		Postural assessment	COP sway area, *p* values	TBI				Non-TBI		
							A significant effect of number of mTBI was found in the postural assessment (*p* = 0.002). Participants with more than one mTBI produced the greatest COP sway area on DYN-Foam.				Patients with no mTBI history produced greater COP sway area on EC-Foam, EO-Foam, and EO, EC, DYN Firm.		
(**f**)													
**Sr.** **No**	**Study Duration**		**VR System**		**Outcomes**	**Participant groups**							
[34]	1 session; clinical tests = 1.5 h and CAREN = 2 h		Computer-Assisted Rehabilitation Environment (CAREN) system (Non-Immersive)		Assessment	Metric	TBI+ Healthy participants						
					Balance Evaluation Systems Test (BESTest)	N/A	Insensitive and non-specific						
					Berg Balance Scale (BBS)	N/A	Insensitive and non-specific						
					Dynamic Gait Index (DGI)	N/A	Insensitive and non-specific						
					High-Level Mobility Assessment Tool (HiMAT)	N/A	Insensitive and non-specific						
					Activities-Specific Balance Confidence (ABC) Scale	N/A	Results not presented clearly						
					Sensorimotor Perturbations (standing and walking)	N/A	Discriminative capabilities: Standing avg. ≈ 0.65 Walking avg. ≈ 0.90						
					Dix–Hallpike Maneuver	N/A	Results not presented clearly						
[35]	1 session		Wii Balance Board and a large flat screen (Non-Immersive)		Assessment	Metric	TBI+ Healthy participants						
					Virtual Environment TBI Screen (VETS) COP sway area	Virtual Environment TBI Screen	Accuracy: 91.0% ROC curve with AUC = 0.865, *p* < 0.001						
						Neurocom Sensory Organization Test	Accuracy: 84.0% ROC curve with AUC, *p* = 0.034						
[36]	1 session		Head Rehab VR System (Non-Immersive)		Assessment	Metric	TBI+ Healthy participants						
					The Balance Error Scoring System (BESS)	Sensitivity, Specificity, AUC	Sensitivity: 85.7% Specificity of 87.8% (cutoff score = 8.25). The AUC = 0.862 (95% CI; 0.767–0.958)						
[37]	1 session		Wii Balance Board, 60″ (75 cm high × 134 cm wide) television (Non-Immersive)		Assessment	Metric	TBI+ Healthy participants						
					DYN-Firm, EO-Foam, EC-Foam, DYN-Foam)	A single forward conditional regression model	Accuracy: 94.4% AUC = 0.998, *p* < 0.001 Sensitivity: 100% Specificity: 93.1%.						
					Balance Error Scoring System	(BESS)	r = –0.15, *p* = 0.21						
[38]	1 session		Wii Balance Board [WBB], 60-inch [75 cm high × 134 cm wide] television (Non-Immersive)		Assessment	Metric	TBI+ non-TBI						
					Virtual Environment TBI Screening (VETS)	(COP sway area, conditions = EO-Firm, EC-Firm, DYN-Firm, EO-Foam, EC-Foam, DYN-Foam)	The mTBI+ group had greater COP sway area during DYN-Foam than the mTBI- group. Across all VETS conditions, the mTBI1+ group generally had increased COP sway compared to the mTBI1 and mTBI- groups.						
[39]	4-week program		Computer-Assisted Rehabilitation Environment (CAREN) system (Non-Immersive)		Assessment	Metric	TBI						
					Balance Balls VE	AUC; *p* value	Balance Balls AUC = 0.618 (*p* = 0.007).						
					Balance Cubes VE (Static and PM)	AUC; *p* value	Balance Cubes—Static AUC = 0.664 (*p* < 0.001). Balance Cubes—PM AUC = 0.688 (*p* < 0.001).						
[40]	1 session		BioVRSea (Immersive)		Assessment	Metric	TBI+ non-TBI						
					COP sway area	Accuracy	Using machine learning and combining SCAT5 and BioVRSea parameters can classify concussion and non-concussion with an accuracy of up to 95.5%.						

B* = Baseline; PT* = Post-treatment (Within 1 week of completing final training session); F* = Follow-up (4 weeks after final session). 6 weeks* = Baseline to 6 weeks; 12 weeks* = Baseline to 12 weeks; 24 weeks* = Baseline to 24 weeks; B** = Baseline; PT** = Post-treatment (after 6 weeks). B* = Baseline. B* = Baseline. B* = Baseline; I* = Intervention phase; R* = Retention phase. B* = Baseline; PT* = Post-treatment.

**Table 3:** Demographic information of participants from all studies.

Sr. No	Type of Study	Total Studies	TBI Sample Size	Gender (TBI)		Age (TBI) (Mean SD)
				Male	Female	
1	Randomized Controlled Trials	3	115	75	40	41.4 ± 2.77
2	One-Arm Experimental Study	1	30	N/A	N/A	20.2 ± 1.46
3	Retrospective Studies	2	30	13	18	55.4 ± 0.2
4	Case Studies	2	2	2	0	47.5 ± 0
5	Feasibility/Usability Study	1	2	N/A	N/A	N/A
6	Cohort Study	1	14	N/A	N/A	25.95 ± 4.48
7	Diagnostic (Validation) Studies	7	334	220 *	73	39.61 ± 7.0

* Please note that not all studies provided a gender breakdown of their participants.

## 4. Discussion

This review investigated the treatment of balance deficits due to traumatic brain injuries using VR therapies. VR systems are computer-based processes that provide a simulated environment and allow a person to respond and interact with this environment in real time. VR systems are believed to be better suited to provide the critical components of neural plasticity to bolster functional recovery outcomes in individuals with neurological conditions. Functional recovery after brain injury is heavily driven by neural plasticity, which is the adaptive capacity of the central nervous system to change in response to experience. In addition to taking advantage of the above principles in simulated media, VR also enriches training environments by engaging sensory, cognitive, and perceptive motor pathways. Therefore, VR-based rehabilitation interventions are generally believed to have a larger positive impact compared to conventional therapy alone. However, well-designed RCTs with a larger sample size are required in TBI patients to truly evaluate the potential of VR-based balance interventions in this population.

The results were mixed in terms of the effectiveness of treating balance and mobility deficits post-TBI utilizing VR therapies compared to the standard of care or other traditional therapy. Due to the relatively low number of RCT studies (N = 3), a firm conclusion could not be made on the effectiveness of VR therapies. Stemming from the fact that very few RCTs exist covering this topic, the papers found only had “fair” methodological quality. Only 115 patients with TBI were involved across the three RCTs surveyed in this review. This shows the lack of large-scale studies evaluating the effectiveness of VR technology to improve balance deficits in this population. While there is growing evidence for improvement in attention, balance, and functional mobility as a result of VR-based treatment for neurological conditions, including patients with stroke, multiple sclerosis, and Parkinson’s disease, this review found mixed and low evidence in TBI patients. In addition, seven out of the 17 studies found were diagnostic (validation) studies that tested whether various VR systems could distinguish patients with TBI from those without TBI. They did not investigate how successful VR therapies were in treating balance deficits in TBI patients. Thus, although VR systems could potentially be a useful diagnostic tool for rehabilitation, there is limited evidence of VR therapies in patients with TBI.

Study results could not be compared across studies as study methodologies varied on VR technologies, training sessions, and outcomes used to assess balance. This review found that most studies using VR-based interventions to improve balance post-TBI used non-immersive VR technology. The Wii balance board and Xbox Kinect were popular among the non-immersive VR technologies, while the Oculus Rift was popular among the immersive technologies surveyed in this review. However, only two RCTs were conducted using the Xbox Kinect while no RCTs used either the Wii balance board or the Oculus Rift/Go. Additionally, there was no evidence of the number of training sessions for VR or the length of each session. None of the reviewed studies presented information regarding their choice of intervention length or number of sessions. The number of training sessions varied from one session to 48 sessions across the studies reviewed. This emphasizes the need for future research to determine dose–response characteristics of VR technology used for balance rehabilitation in patients with TBI. We recommend that all future studies use standard balance outcomes to enable comparison between study results.

This review also observed that significantly fewer females were studied in this population than men. Only 40 females were involved compared to 75 males across RCTs, while only 73 females were included compared to 220 males in validation studies. Future studies need to have an equal distribution of males and females and look at sex-related differences when evaluating the effectiveness of VR-based balance rehabilitation in TBI patients.

A potential limitation of this review is that studies focusing on older adults or including children were excluded. This could have excluded a vital population that is frequently affected by traumatic brain injuries. However, evidence from older adults and children cannot be compared to the evidence pool in the adult population. Another potential limitation is that due to limited studies, different types of VR systems could not be analyzed separately to determine if certain systems led to better outcomes. Finally, many of the studies found had low patient populations, so the strength of their findings is limited. The lack of literature, especially RCTs, on VR therapies to treat balance deficits in TBI patients makes it very difficult to conduct a proper meta-analysis. Hopefully, more RCTs will be conducted to evaluate the effectiveness of VR-based therapies to treat balance deficits.

## 5. Conclusions

This study investigated the evidence for the use of VR-based therapy to improve mobility and balance in individuals with TBI. This scoping review found that most studies using VR therapy in this population were basic validation studies using different VR equipment. There was no particular VR equipment that was popularly used in studies. The evidence on the effectiveness of VR-based therapy for improving balance deficits in this population is weak, and more RCT studies with large sample sizes and equal representation of females need to be conducted to better understand the effectiveness of VR-based therapy in individuals with TBI.

## Data Availability

No new data has been generated as part of this review. Data used for analysis in this review will be made available upon request.

## Supplementary Materials

The following supporting information can be downloaded at https://www.mdpi.com/article/10.3390/brainsci14050429/s1. Detailed PEDro scale analysis for studies is provided in Supplementary Materials.
