# Supplementary material for: Virtual Reality-Based Interventions to Improve Balance in Patients with Traumatic Brain Injury: A Scoping Review
Source: Brain Sci. 2024 Apr 26;14(5):429. doi: 10.3390/brainsci14050429 (PMC11119161; doi:10.3390/brainsci14050429)
Supplement: Supplementary file 1 [file brainsci-14-00429-s001.zip › brainsci-2959426-supplementary.pdf]

Detailed PEDro scale rating for selected studies.

Sr. No	Date Published	Eligibility criteria were specified (This question is not considered for total score)	Subjects were randomly allocated to groups	Allocation was concealed	The groups were similar at baseline regarding the most important prognostic indicators	There was blinding of all subjects	There was blinding of all therapists who administered the therapy	There was blinding of all assessors who measured at least one key outcome	Measures of at least one key outcome were obtained from more than 85% of the subjects initially allocated to groups	At least one key outcome was analysed by "intention to treat"	Between-group statistical comparisons are reported for at least one key outcome	Point measures and measures of variability for at least one key outcome	Total score
[24]	2022	Y	Y	Y	Y	N	N	Y	Y	Y	Y	Y	8
[25]	2019	Y	Y	Y	Y	N	N	Y	Y	Y	Y	Y	8
[26]	2017	Y	Y	Y	Y	N	N	N	Y	Y	Y	Y	7
[27]	2019	Y	N	N	Y	N	N	N	Y	N	N	Y	3
[28]	2019	Y	N	N	N	N	N	N	Y	N	Y	Y	3
[29]	2022	Y	N	N	Y	N	N	N	Y	Y	Y	Y	5
[30]	2017	Y	N	N	N	N	Y	N	Y	Y	N	Y	4
[31]	2023	N	N	N	N	N	N	N	Y	Y	N	Y	3
[32]	2022	N	N	N	N	N	N	N	N	Y	Y	Y	3
[33]	2018	Y	N	N	N	N	N	N	Y	N	Y	Y	3
[34]	2020	**Y**	N	N	**Y**	N	N	N	**Y**	**Y**	**Y**	**Y**	5

Detailed PEDro scale rating for selected studies.

[35]	2016	Y	N	N	Y	N	N	N	Y	Y	Y	Y	5
[36]	2016	Y	N	N	Y	N	N	N	Y	Y	Y	N	4
[37]	2017	Y	N	N	N	N	N	N	Y	Y	Y	Y	4
[38]	2022	Y	N	N	Y	N	N	N	Y	Y	Y	Y	5
[39]	2017	Y	N	N	N	N	N	Y	Y	Y	Y	Y	5
[40]	2022	N	N	N	N	N	N	N	Y	Y	Y	Y	4

**Y=Yes; N=No
